# Editorial: Environmental Effect on Neuroinflammation and Neurodegeneration

**DOI:** 10.3389/fncel.2022.935190

**Published:** 2022-06-21

**Authors:** Monica Renee Langley, Srikant Rangaraju, Adwitia Dey, Souvarish Sarkar

**Affiliations:** ^1^Department of Physical Medicine and Rehabilitation, Rehabilitation Medicine Research Center, Mayo Clinic, Rochester, MN, United States; ^2^Department of Neurology, Emory University, Atlanta, GA, United States; ^3^Department of Pathology, Brigham and Women's Hospital, Harvard Medical School, Boston, MA, United States

**Keywords:** neuroinflammation, neurodegeneration, genetic, GWAS, gene-environment interactions

## Introduction

Neurodegenerative disorders like Parkinson's disease and Alzheimer's disease, among others, are characterized by the loss of a specific subset of neurons and glial activation. Genome-wide associated studies (GWAS) have identified various genetical risk factors, and environmental risk factors have been found through epidemiological studies. For example, metals, diet, occupational exposure to pesticides, and pollution, among others, have been shown to play a key role in the manifestation of neurodegeneration and neuroinflammation. Furthermore, interactions between genetic and environmental factors play a key role in these disease etiologies.

Though environmental factors have been acknowledged in the etiology of various neurodegenerative disorders, not much is known about the intracellular mechanisms involved in these key gene-environment interactions. A clear understanding of these mechanisms will not only help to understand these diseases better but may also be used for lead identification in drug discovery. With recent advancements of GWAS and epidemiological studies, mechanistic studies in cell culture and animal models can be linked back to the human population. A series of recent studies delved into the potential contribution of various environmental factors in regulating neuroinflammation and neurodegeneration in animal models and human populations.

## Neuroinflammation

Previous studies have found conflicting results on the effect of season on relapses in multiple sclerosis (MS) patients; however, winter was associated with higher relapse rates in Jeddah, Saudi Arabia in the study by Makkawi et. al. Further, this was linked to low vitamin D levels during winter months. The multifactorial effect of seasons and vitamin D, along with environmental and dietary habit changes during different times of year, should be pursued in future studies in larger populations including other geographic regions with higher prevalence and with different racial demographics as well. Understanding seasonal variation in environmental contributors to MS could help to reduce overall disease incidence as well as molecular consequences.

A potential contributor to the persistent neuroinflammation associated with Gulf War Illness was identified using a rat model. Briefly, Attaluri et al. demonstrated that a brain-specific increase in leukotriene (LT) signaling occurs along with increased pro-inflammatory cytokine levels following chronic exposure to GWI-related chemicals (pyridostigmine bromide, DEET, and permethrin) and moderate stress. Brain-derived extracellular vesicles (EVs) found in blood were able to trace this increase in LT signaling noninvasively, suggesting a potential biomarker. Another study by Fernades et al. used a model of chronic unpredictable stress to identify the essential role of iNOS on adult neurogenesis in the mouse hippocampus.

Relating to potential occupational exposures, agricultural contaminants from the animal production industry include organic dust and are known to contribute to respiratory symptoms in affected individuals. The study by Massey et al. expands this to the central nervous system by demonstrating neuroinflammatory changes in multiple brain regions as well as motor deficits and olfactory impairment. Importantly, some of these effects were mitigated when the mice were administered mitoapocynin, a mitochondrially targeted antioxidant that has been shown to protect against key pathological features in multiple models of Parkinson's disease (PD), a neurodegenerative disorder characterized by motor deficits and with an etiology known to involve gene and environmental factors.

## Neurodegeneration

Since air pollution is a risk factor for dementias, including Alzheimer's disease (AD), Patten et al. investigated the effect of traffic-related air pollution (TRAP) on the TgF344 rat model of AD and found that early inflammatory responses in the hippocampus are sex- and age-dependent, but are not reflected by the serum cytokine profiles, highlighting the limitation of their prognostic value in this case. Neuroinflammation is hypothesized to link exposures to inhaled pollutants and the interacting deleterious pathways resulting in neurodegenerative diseases. To discern and validate biomarkers of progressive neurodegeneration in amyotrophic lateral sclerosis (ALS), bioinformatics and other approaches were employed by Li et al. Importantly, their group found that DHRS4 is upregulated in a widely used mouse model of ALS and that this increase involves the immune system, specifically including activation of the complement cascade.

A model of organophosphate nerve agent (OPNA) soman exposure was evaluated in rats for the development of seizure activity and demonstrated sex-specific effects. This paradigm more closely models civilian exposure by not utilizing military-relevant pre-treatments and recapitulates relevant spontaneous reoccurring seizures and neuropathology. Similarly, another organophosphate, diisopropylfluorophosphate (DFP), was used to demonstrate the ability of saracatinib, an Src/Fyn tyrosine kinase inhibitor, to ameliorate the number of animals with spontaneous recurrent seizure activity and neurodegeneration.

Overall, this issue covers many environmental factors that can affect neuroinflammatory and neurodegenerative disorders, ranging from seasonal effects and nutritional status to occupational hazards and civilian exposure to nerve agents. The contributions of environmental factors alone, or in combination with genetics, sex, and other environmental factors, across the lifespan on neuroinflammation and neurodegenerative diseases highlight the importance of maintaining tight control over these molecular processes to prevent progression and maximize repair potential. This underscores the need for multidisciplinary exosome studies to be considered along with genomic studies in reaching the goals of novel therapeutic strategies and individualized medicine for these neurological disorders. Moreover, the new mechanisms identified in the abovementioned studies could be further developed as new therapeutic targets for their respective neurological disorders in future studies.

## Author Contributions

ML and SS were guest associate editors of the Research Topic and wrote the paper text. SR and AD were guest associate editors of the Research Topic and edited the text. All authors contributed to the article and approved the submitted version.

## Conflict of Interest

The authors declare that the research was conducted in the absence of any commercial or financial relationships that could be construed as a potential conflict of interest.

## Publisher's Note

All claims expressed in this article are solely those of the authors and do not necessarily represent those of their affiliated organizations, or those of the publisher, the editors and the reviewers. Any product that may be evaluated in this article, or claim that may be made by its manufacturer, is not guaranteed or endorsed by the publisher.

